# Altered functional properties of the codling moth Orco mutagenized in the intracellular loop-3

**DOI:** 10.1038/s41598-021-83024-3

**Published:** 2021-02-16

**Authors:** Yuriy V. Bobkov, William B. Walker III, Alberto Maria Cattaneo

**Affiliations:** 1 Whitney Laboratory, Center for Smell and Taste, and McKnight Brain Institute, University of Florida, Gainesville, FL USA; 2 Department of Plant Protection Biology, Chemical Ecology Unit, Swedish University of Agricultural Sciences, Alnarp, Sweden

**Keywords:** Ion channels, Membrane proteins, Patch clamp, Voltage clamp, Fluorescence imaging, Bioinformatics, Expression systems, Phylogeny, Olfactory receptors, Receptor pharmacology, Entomology

## Abstract

Amino acid substitutions within the conserved polypeptide sequence of the insect olfactory receptor co-receptor (Orco) have been demonstrated to influence its pharmacological properties. By sequence analysis and phylogenetic investigation, in the Lepidopteran subgroup Ditrysia we identified a fixed substitution in the intracellular loop-3 (ICL-3) of a conserved histidine to glutamine. By means of HEK293 cells as a heterologous system, we functionally expressed Orco from the Ditrysian model *Cydia pomonella* (CpomOrco) and compared its functional properties with a site-directed mutagenized version where this ICL-3-glutamine was reverted to histidine (CpomOrco^Q417H^). The mutagenized CpomOrco^Q417H^ displayed decreased responsiveness to VUAA1 and reduced response efficacy to an odorant agonist was observed, when co-transfected with the respective OR subunit. Evidence of reduced responsiveness and sensitivity to ligands for the mutagenized Orco suggest the fixed glutamine substitution to be optimized for functionality of the cation channel within Ditrysia. In addition, contrary to the wild type, the mutagenized CpomOrco^Q417H^ preserved characteristics of VUAA-binding when physiologic conditions turned to acidic. Taken together, our findings provide further evidence of the importance of ICL-3 in forming basic functional properties of insect Orco- and Orco/OR-channels, and suggest involvement of ICL-3 in the potential functional adaptation of Ditrysian Orcos to acidified extra-/intracellular environment.

## Introduction

The odorant receptor co-receptor, Orco, is a unique transmembrane protein, expressed in most of the olfactory sensory neurons (OSNs) of insect antennae^1–3^ and is highly conserved in sequence and function across all insects^4,5^.

The role of Orco is renowned for forming tetrameric complexes^6^ working as ligand-gated cation channels, both homomeric and heteromeric with odorant receptor (OR) subunits^2,7–13^, permeable to Ca^++^ and monovalent cations such as Na^+^ and K^+^^14–18^. Apart from working as cation channels, other studies suggest involvement of Orco + OR heteromers in signal transduction downstream of odorant binding, in which a metabotropic response presumably involves either activation of adenylyl cyclase stimulating Gs proteins and protein kinase C (PKC)-dependent Orco phosphorylation or ORs coupling to Gq-proteins which activate phospholipase Cβ (PLCb)^19–21^. In addition, Orco may play a structural role in OSNs, working as a chaperone in the trafficking of OR-subunits to the plasma membranes by compartmentalizing ORs to the dendritic segments of chemosensory neurons^2,13,22,23^.

Orco, per se, is perhaps the most important subunit for insect olfactory reception; being the most broadly expressed and conserved transmembrane protein among all insect orders, independently of their species-specific OR-repertoires^24,25^. Expression of Orco in insect OSNs is fundamental for the generation of a functional olfactory system, and disruption of its expression can dramatically impair behavioural responses triggered by odorant sensing^19,26–30^. Understanding evolutionary and pharmacological aspects of Orco activation is essential to shed light on mechanisms underlying insect olfactory sensory function.

In search of a novel class of repellents acting on all insect OSNs at once, pharmacological investigations addressed Orco as a possible target for insect chemosensory disruption. A class of synthetic compounds derived from 2-(4-ethyl-5-(pyridin-3-yl)-4H-1,2,4-triazol-3-ylthio)-N-(4-ethylphenyl)acetamide, named as VUAAs^16^, have been identified as agonists of olfactory co-receptors^31–33^. In parallel, drugs active as Orco-inhibitors were discovered among the amiloride derivatives, such as the 5-(N-methyl-N-isobutyl) amiloride (MIA)^34,35^.

These compounds serve as tools to investigate kinetic and pharmacological properties of Orco homomers and Orco + OR heteromers. For example, the use of VUAAs unveiled aspartate residues at position 357 and 466 of the *D. melanogaster* co-receptor being responsible for a decreased sensitivity to VUAA if mutagenized^36^. In addition, mutagenesis studies have implicated cysteine residues (C409, C429 and C449) in increasing VUAA-sensitivity, while mutagenesis of others cysteines (C228 and C446), decreased VUAA-sensitivity^37^. With the use of the VUAA-agonists, other investigations conducted on the *Bombyx mori* Orco unveiled the importance of additional residues involved in the ion channel-gating and activation, such as tyrosine-464 influencing current–voltage relationships and K^+^-selectivity^38^. More recently, a naturally-occurring VUAA-insensitive Orco was described for the Hessian fly *Mayetiola destructor*: the basis of the limited sensitivity of this Orco to VUAA-agonists is attributed to a wide distribution of critical amino acid residues along the full-length polypeptide sequence that collectively contribute to ligand-binding^39^.

Together with amino acid residues of importance for Orco functionality, intracellular loops (ICLs) located on the cytosolic side of the plasma membranes of OSNs have been demonstrated to be at the base of olfactory-subunit interactions for the formation of the Orco/OR heteromers^6,12,13^. Evidence has been reported on the involvement of the ICL-3 domain of Orco and of invariable C-terminal residues within ORs in subunit-subunit interactions^40–45^. In accordance, additional findings identified conserved motifs within the ICL-3 involved in the Orco protein heteromerization^46^. Indeed, it has been suggested that conserved residues within domains proximal to the Orco C-terminus mediating the functional interactions of Orco and OR subunits maintained the primary function of the co-receptor since the divergence of Lepidopteran and Dipteran lineages^4,6,41,44^. The importance of conserved residues proximal to the C-terminus in the overall function of Orco^47^ motivates investigations to validate possible influences of single amino acid residues, in terms of Orco/OR heteromerization, odorant binding of the OR-subunit, opening of a ligand-gated cation channel and, possibly, metabotropic interactions with other proteins of the olfactory signal transduction machinery^18–21,24,25^.

Analysis of protein sequence of insect Orcos shows that most Lepidoptera representatives contained a glutamine residue (Q) in substitution of a highly conserved histidine residue of the ICL-3; named here ICL-3 H to Q. After its appearance in Lepidoptera, this substitution became fixed only among Neolepidopterans of the subgroup Ditrysia. Apart from Lepidoptera, this substitution also occurred among a limited number of insects belonging to different orders. Interest in this amino acid substitution was based on evidence from previous investigations demonstrating the importance of its adjacent amino acid residues in Orco-VUAA-binding^37,39^. Further interest was raised by evidence demonstrating the involvement of other residues adjacent to this substitution in protein–protein interaction^47–49^, such as a tryptophan residue conserved among sequences of all insects’ Orco and OR subunits^46^.

To test a possible role of this amino acid substitution in the kinetic and pharmacological properties of a Ditrysian Orco, we generated a site-directed mutagenized version of the *Cydia pomonella* Orco (CpomOrco), which we heterologously expressed and functionally characterized in HEK293-cells by standard calcium imaging and patch-clamp recordings. Site-directed mutagenesis (CpomOrco^Q417H^) substituted in the highly conserved histidine rather than glutamine at the position presenting the amino acid substitution found within Ditrysia. The mutagenization was intended to isolate the effect of this amino acid locus within the Ditrysian Orco sequence, simulating the polypeptide sequence of a non-Ditrysian Orco, which is commonly provided with an ICL-3 histidine. Basic kinetic and pharmacological studies were performed testing activation by VUAA1 and by an odorant ligand when Orco was co-expressed with a CpomOR-subunit, and comparing results with the wild type CpomOrco.

The conjugate acid of the imidazole side chain in histidine has a pKa of approximately 6.0, which is within the physiologic range; we hypothesized that acidification of intracellular pH may lead to protonation the histidine imidazole side chain, potentially influencing sterical and binding properties of ICL-3. To test this hypothesis, we compared functional properties between physiologic and acidic pH conditions for both the wild type and the mutagenized CpomOrco, heterologously expressed in HEK293 cells.

## Results

### Analysis of the ICL-3 domain of insect olfactory co-receptors

**Figure 1:**
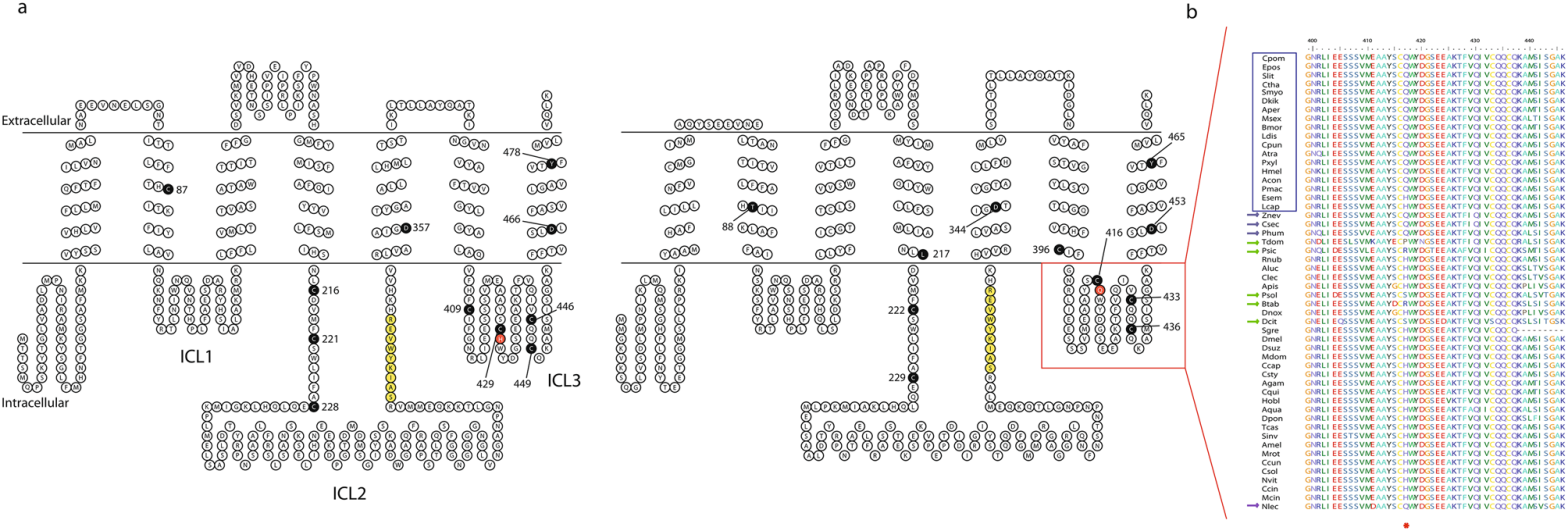
Analysis of the Orco polypeptide sequence: topology and ICL-3. (**a**)—topological representation of Orco, between *D. melanogaster* (left) and *C. pomonella* (right). Numbered black residues: amino acids with possible influences for DmelOrco VUAA-binding and sensitivity [cysteines, Turner et al.^37^; aspartates 357 and 466^36^, the respective position on DmelOrco related to the *B. mori* tyrosine 464^38^, for which mutagenization alters current–voltage relationships, and Potassium selectivity (tyrosine 478), and their respective positions on CpomOrco (right)]. Yellow residues: Calmodulin binding sites (“SAIKYWVER”^50^). Red residues: ICL-3 histidine (left) and the respective substitution on CpomOrco (Q, right). Red square: ICL-3 alignment based on Topcons predictions of Dmel- and CpomOrco. (**b**)—sequence alignment of the ICL-3 of Orco amino acid sequences among insects of the Supplementary Dataset file. Acronyms for insect names are given based on Table 1. Blue square: Lepidopterans; blue arrows: non-Lepidopterans insects provided with the H-Q substitution; green arrows: non-Lepidopteran insects provided with other types of substitutions at the H417 position (red asterisk). Numbers on the top: positions of amino acid residues based on the polypeptide sequence of CpomOrco.

**Figure 2:**
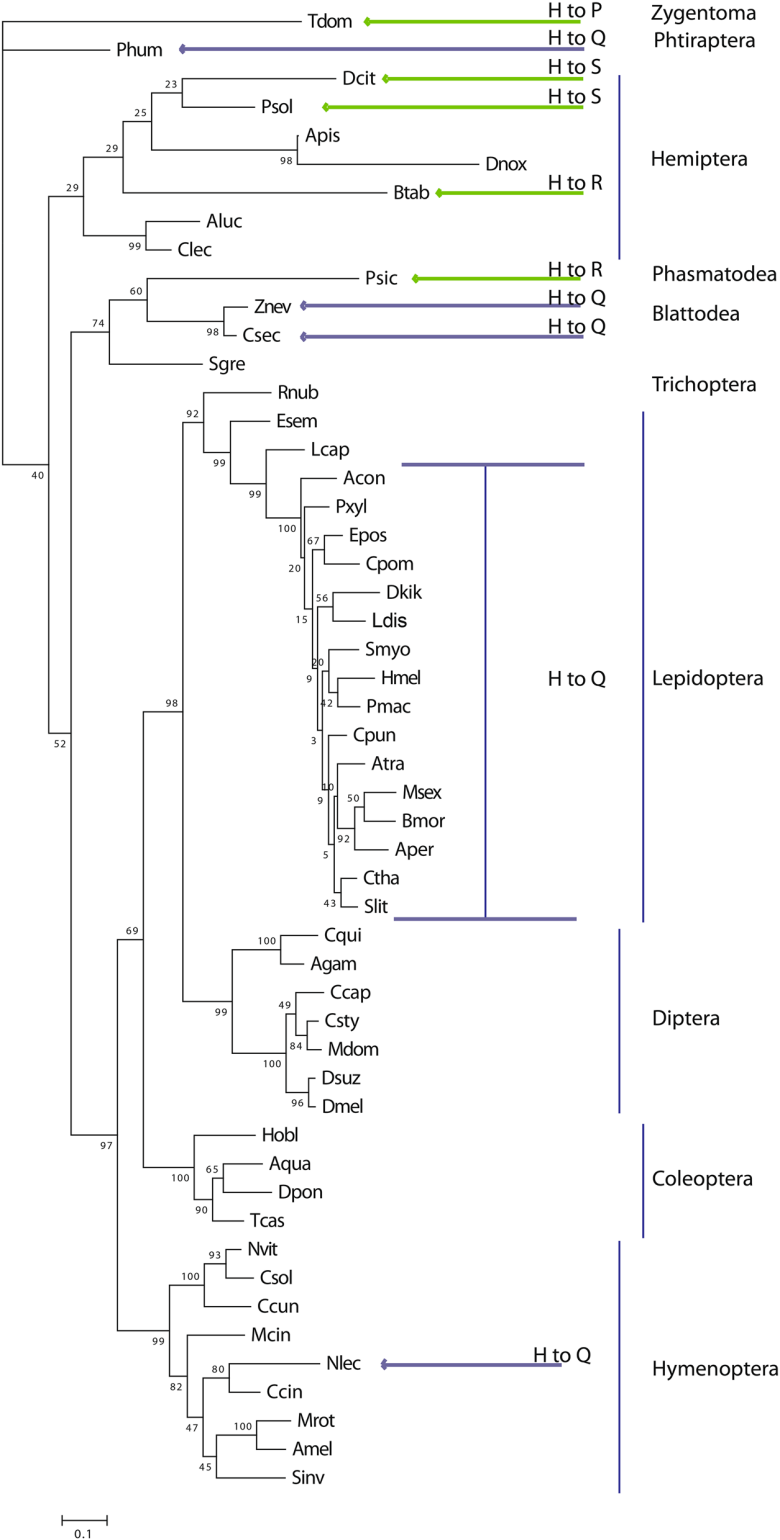
Maximum likelihood phylogenetic tree of Orco sequences. Unrooted. Includes sequences from Table 1. Node support was assessed with 600 bootstrap replicates. Blue arrows: non-Lepidopteran insects provided with the H-Q substitution; green arrows: non-Lepidopteran insects provided with other types of substitutions at the H417 position.

The alignment of a collection of 52 protein sequences identified a specific amino acidic substitution, glutamine rather than histidine (H to Q), at the position 417 of the polypeptide sequence of *C. pomonella* Orco and of orthologues of most other Lepidopteran representatives. Topologic predictions comparing *C. pomonella* Orco with that of *D. melanogaster*, revealed the position of this residue within the ICL-3 domain of the folded transmembrane protein (Fig. 1). Analysing additional Lepidoptera representatives, sequence alignment demonstrated that this amino acid substitution is shared among Neo-lepidopterans belonging to the subgroup Ditrysia (http://tolweb.org); while the substitution is absent for the non-Ditrysian representative *Lampronia capitella* (Lepidoptera: Prodoxidae) and for the non-Neolepidopteran representative *Eriocrania semipurpurella* (Lepidoptera: Eriocranidae) (Fig. 1b, Supplementary Dataset file). Phylogenetic analysis demonstrated that the H to Q substitution also occurred within insect orders diverged earlier than Lepidoptera (Fig. 2): the human body louse *Pediculus humanus corporis* L. (Phthiraptera: Pediculidae), the eusocial termite *Zootermopsis nevadensis* Hagen (Blattodea: Thermopsidae), the West Indian dry-wood termite *Cryptotermes secundus* Hill (Blattodea: Kalotermitidae) and the sawfly *Neodiprion lecontei* Fitch (Hymenoptera: Diprionidae).

In addition, amino acid substitutions different from histidine and glutamine have been identified for more primitive insects: *Thermobia domestica* Pakard (Tysanura: Lepismatidae) has proline (H to P); *Phenacoccus solenopsis* Tinsley (Hemiptera: Pseudococcidae) and *Diaphorina citri* Kuwayama (Hemiptera: Psylloidea) have serine (H to S); *Bemisia tabaci* Gennadius (Hemiptera: Aleyrodidae) and *Psyllum siccifolium* L (Phasmatodea: Phyllidae) have arginine (H to R) (Figs. 1b, 2).

In total, among analysed sequences from non-Ditrysian insects, 27 out of 36 (75%) contained histidine at the specified residue, while different amino acid substitutions were identified for 25% of the sequences.

### Functional expression of CpomOrco and CpomOrco^Q417H^

Functional expression of CpomOrco and CpomOrco^Q417H^ variants in HEK293A cells was validated by application of VUAA1. An evaluation of the expression level in HEK cell preparations was based on a comparison between the VUAA1-responsiveness and the number of cells positive for EBFP-fluorescence, given the regulation of both Orco- and EBFP-expression by the same promoter (CMV, see methods) (Supplementary Table S1). We found that when HEK293A cells were transfected with CpomOrco and EBFP, nearly all EBFP-positive cells (33.05–40.94%, relative to total number of visible cells) were sensitive to 800 µM VUAA1 (30.99–35.06%). Contrarily, a similar percentage of EBFP-positive cells was identified when cells were transfected with CpomOrco^Q417H^ and EBFP (38.55–40.66%) but a lower percentage of cells responded to 800 µM VUAA1 (17.03–18.44%). In a parallel set of experiments, HEK293A cells were co-transfected with the CpomOrco variants, CpomOR6a and EBFP. For these experiments, the percentage of EBFP-positives was 30.70–31.13% versus 48.34–49.71% VUAA-responsive, in the cells co-transfected with CpomOrco. On the other hand, CpomOrco^Q417H^ co-transfection yielded similar percentages of EBFP-positives (27.66–29.87%) but greatly reduced number of VUAA1 sensitive cells (8.41–10.33%).

**Figure 3:**
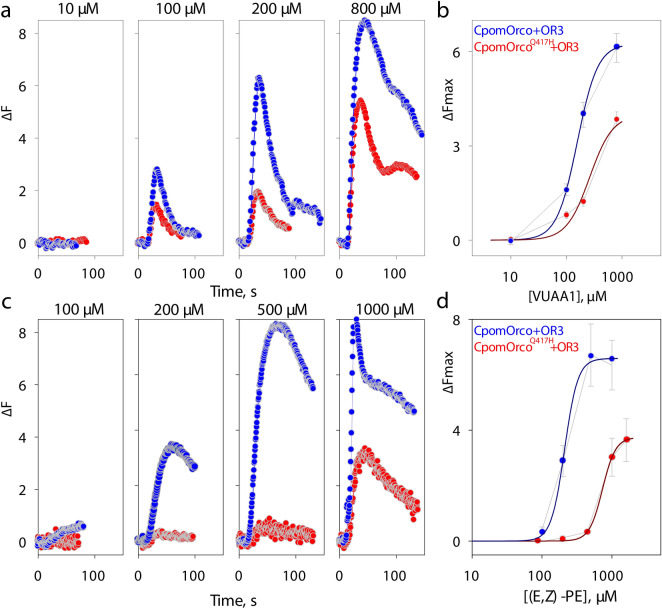
Comparison of HEK cell responses expressing wild-type and mutagenized form of Orco. (**a**)—Universal Orco agonist, VUAA1, elicits dose-dependent Ca^++^_i_ increase in HEK293A cells expressing either CpomOrco (blue) or CpomOrco^Q417H^ (red). (**b**)—VUAA1 concentration dependencies. Data points represent the mean response amplitudes (± SE). Data were fit to a Hill equation for CpomOrco (blue smooth line) or CpomOrco^Q417H^ (red smooth line) respectively, providing maximum responses of ~ 6.3 ± 0.1 and ~ 3.9 ± 1.3 ∆F, EC50s of ~ 157.1 ± 3.58 and ~ 261.8 ± 165.6 µM and Hill coefficients of ~ 2.4 ± 0.1 and ~ 2.1 ± 1.9; total number of cells analysed: N = 123 and 94. (**c**)—Effects of Pear ester on the activity of CpomOrco+OR3 (blue) and CpomOrco^Q417H^+OR3 (red) heteromers. (**d**)—Pear ester concentration dependencies. Data points represent the mean response amplitudes (± SE) of cells from at least three experiments. Data were fit to a Hill equation providing the following parameters: maximum responses of ~ 6.6 ± 0.3 and 3.7 ± 0.1 ∆F; EC50s of ~ 210 ± 14.2 and ~ 733.5 ± 26.9 µM; Hill coefficients of ~ 4.6 ± 2.4 and ~ 4.6 ± 0.4, for CpomOR3/CpomOrco (blue smooth line) or CpomOR3/CpomOrco^Q417H^ (red smooth line) respectively. Total number of cells analysed: N = 160 and 262. Traces in A and C represent the mean responses of cells from one experiment. Data in B and D were not normalized. Scales in B and D are different.

Next, we compared VUAA1 dose–response characteristics obtained from HEK293A cells expressing the CpomOrco and CpomOrco^Q417H^ variants. Application of the agonist (10–800 µM, Fig. 3a) elicited calcium responses in a concentration dependent manner in both cases. Responses of the cells transfected with the mutagenized CpomOrco^Q417H^ receptor were greatly reduced (~ 6.3 vs. ~ 3.9 ∆F, [VUAA1] = 1000 µM, Fig. 3b), perhaps, suggesting lower sensitivity of the mutagenized receptor to VUAA1 (EC50 ~ 261 µM) as compared with wild-type CpomOrco (EC50 ~ 157 µM, Fig. 3b).

To test whether CpomOrco^Q417H^ similarly changes the functionality of Orco+OR complexes, we co-expressed CpomOrco or CpomOrco^Q417H^ with CpomOR3 and used the cognate odorant of CpomOR3 (ethyl (E,Z)-2,4-decadienoate (pear ester)^14,51^) to stimulate the cells (100–1000 µM, Fig. 3c,d). As would be predicted from the VUAA1 experiments, the sensitivity to pear ester in the cell systems co-expressing CpomOR3 with the mutagenized CpomOrco^Q417H^ was associated with higher half-maximal doses indicative of lower sensitivity (EC50_(E,Z)-PE_^C1251^ ~ 733 µM) when compared with the co-expression with the wild-type CpomOrco (EC50_(E,Z)-PE_ ~ 210 µM). Both concentration dependencies were characterized by similar cooperativity coefficients: h_(E,Z)-PE_ ~ 4.6; h_(E,Z)-PE_^C1251^ ~ 4.6), consistent with previously reported estimates^14^.

### pH-sensitivity studies

**Figure 4:**
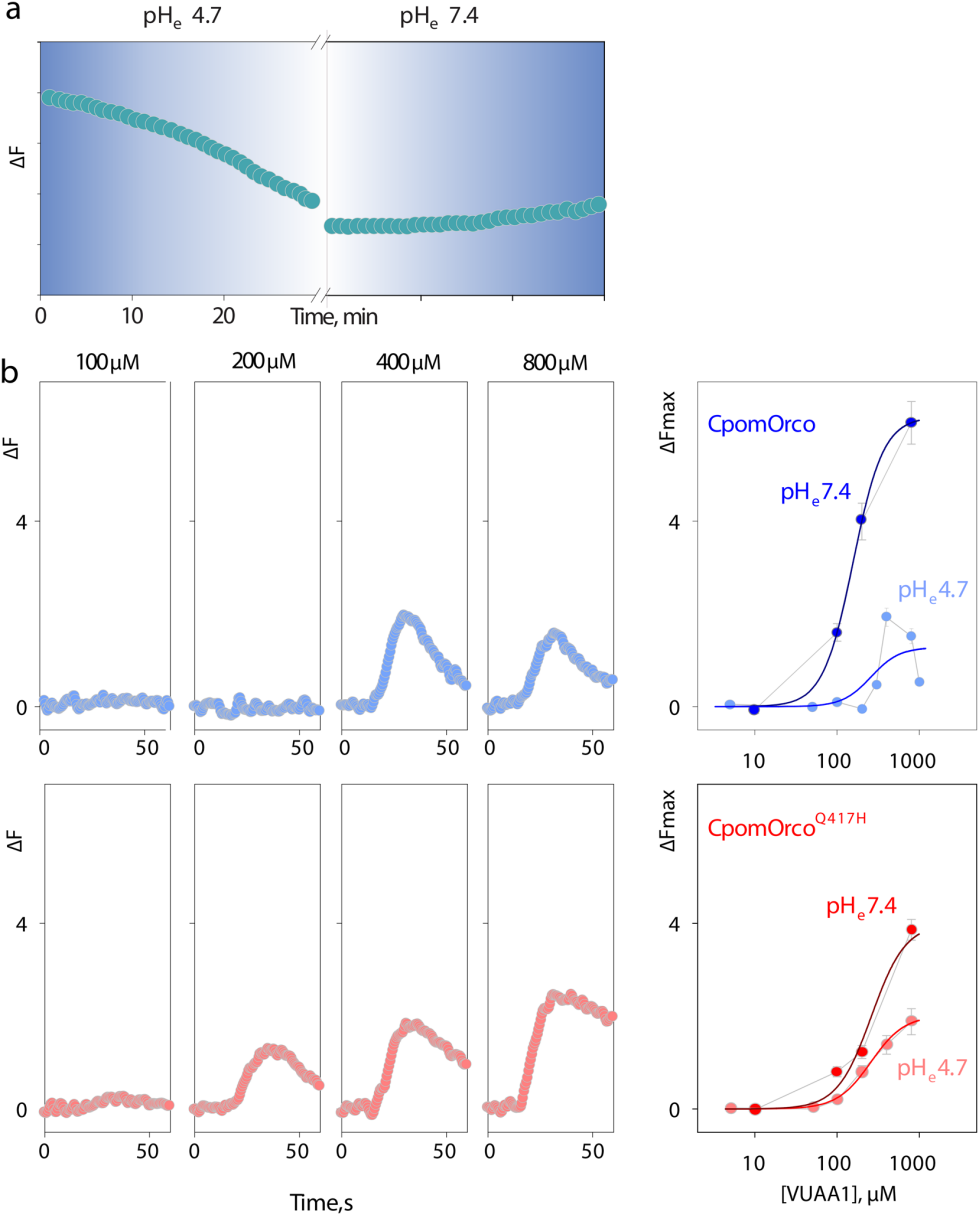
Testing pH sensitivity of HEK cell expressing wild-type and mutagenized form of Orco. (**a**)—Decrease in fluorescence intensity of the pH sensitive probe, BCECF, possibly reflects acidification of cytoplasm in response to low pH extracellular conditions. (**b**)—Comparison of VUAA1 concentration dependencies obtained after 30 min incubation at low pH_e_ for CpomOrco or CpomOrco^Q417H^. Left panels—VUAA1 activated calcium responses. Right panels—VUAA1 concentration dependencies. Data points represent the mean response amplitudes (± SE). Data were fit to a Hill equation with the following parameters: maximum responses of ~ 1.3 ± 0.7 and ~ 1.8 ± 0.2 ∆F; EC50s of ~ 260 ± 187 and ~ 263.3 ± 45.3 µM; Hill coefficients of ~ 2.5 ± 4.3 and ~ 2.2 ± 0.5, for CpomOrco (blue smooth line, n = 84) or CpomOrco^Q417H^ (red smooth line, n = 63) respectively. Constraints were applied to fit greatly scattered data in B. Traces in B (left panels) represent the mean responses of cells from one experiment. Data in B (right panels) were not normalized. Concentration dependencies obtained in physiologically relevant control conditions were taken from Fig. 3.

The histidine imidazole side chain has a pKa of approximately 6.0, suggesting that insect Orco functionality might change depending on physiologically relevant pH changes^52^. We hypothesized that acidification of intracellular pH may cause protonation of the histidine imidazole side chain, potentially influencing sterical and binding properties of ICL-3. To test this hypothesis, we compared functional properties at physiologic and acidic pH conditions for both the wild type and the mutagenized CpomOrco heterologously expressed in HEK293 cells. Generally, extracellular pH changes, if beyond the physiologically relevant pH range (~ 6.8–7.6), would shift intracellular pH with ∆pH_e_/∆pH_i_ ~ 1^53^. To estimate the kinetics of intracellular acidification in response to extracellular pH shift from ~ 7.4 to 4.7 we used a pH-sensitive dye 2′,7′-bis-(2-carboxyethyl)-5-(-6)-carboxyfluorescein (BCECF dye, Fig. 4a). HEK cells loaded with BCECF showed relatively slow decrease in fluorescence intensity during incubation at pH_e_ = 4.7. While the decrease in fluorescence intensity appear to be only partially reversible, the approach allows reliably reducing pH_i_ and qualitatively estimating physiological properties of the receptors at different pH conditions.

We then compared VUAA1 concentration dependencies for CpomOrco or CpomOrco^Q417H^ obtained at acidified intracellular conditions (pH_e_ = 4.7). Interestingly, response amplitudes of CpomOrco to VUAA1 (100–800 µM) appeared to be substantially reduced at low pH (1.3 vs. 6.3, ∆F in control pH conditions), while CpomOrco^Q417H^ appeared to be less susceptible at this condition (1.8 vs. 3.9, ∆F in control pH conditions, Fig. 4b, right panels).

**Figure 5:**
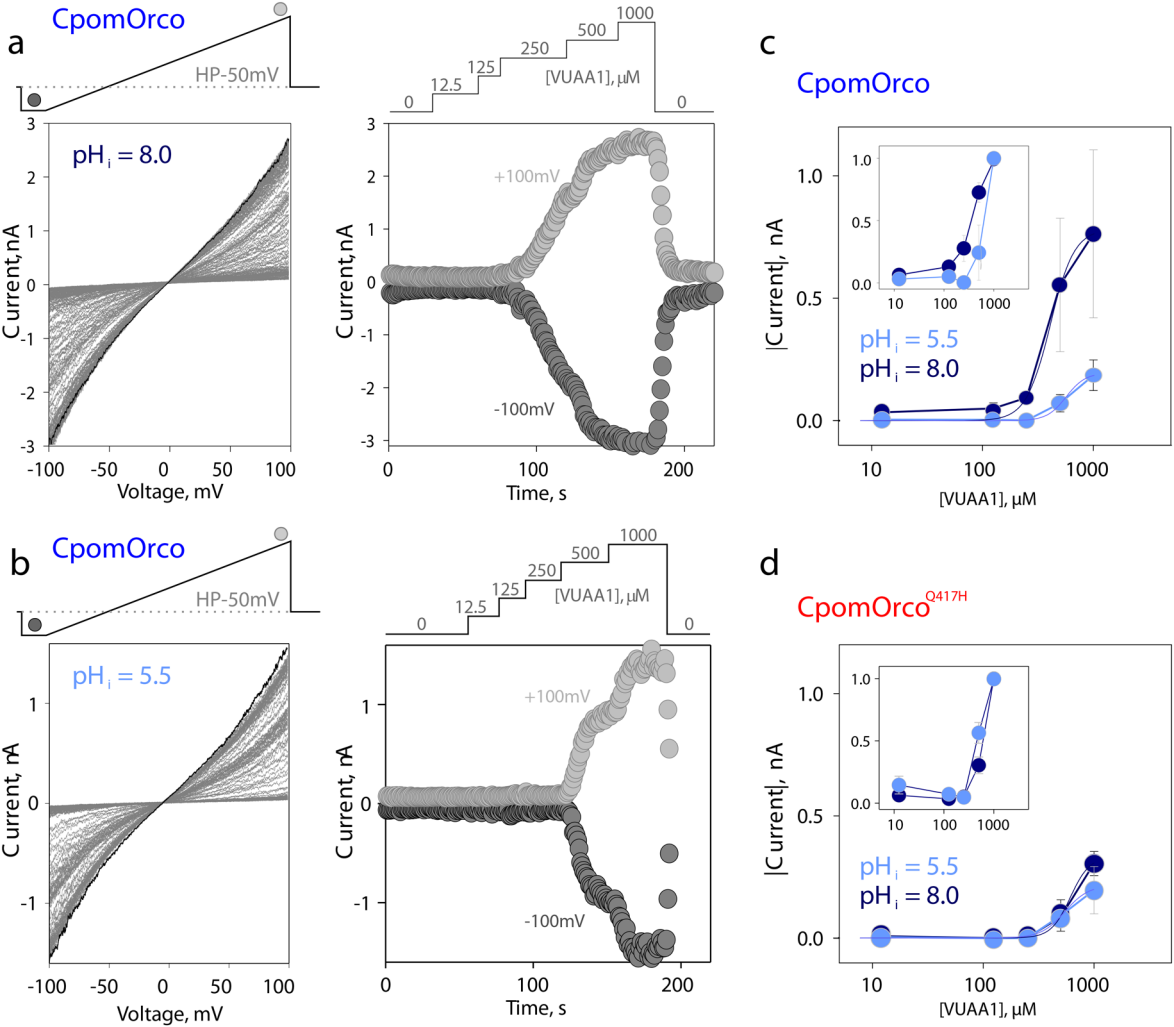
Properties of the wild type CpomOrco and the mutagenized CpomOrco^C1215^ at different pH_i_. (**a**), (**b**)—wild type CpomOrco, whole-cell current–voltage (CV) characteristics at different intracellular pH conditions and comparison of VUAA1 effects. Left: series of current–voltage relationships of HEK293A cells transfected with CpomOrco obtained in the presence of different [VUAA1]. Right: current time courses upon application of different concentrations of VUAA1. Current values were estimated based on CV characteristics (left, respective symbols) at − 100 and ~  + 100 mV. VUAA1 effects were reversible. Note, Current scales and agonist application diagrams in (**a**), (**b**) are different. A (pH_i_ = 8.0) and B (pH_i_ = 5.5) – different cells. The pH of the media (extracellular) was maintained at 7.4. (**c**)—summary of the effects of different pH_i_s on the agonist concentration dependences for CpomOrco. Smooth lines, results of approximation by Hill equation: I_max_ ~ 784 ± 40 pA, K_1/2_ ~ 403 ± 23 µM, h ~ 4 ± 0.6 at pH = 8.0 (dark blue, number of cells sensitive to VUAA1—8 out of 13 tested); I_max_ ~ 196 + -32 pA, K_1/2_ ~ 561 ± 84 µM, h ~ 5 ± 3.9 at pH = 5.5 (light blue, number of cells sensitive to VUAA1—3 out of 4 tested). (**d**)—summary of the effects of different pH_i_s on the agonist concentration dependences for CpomOrco^C1215^. Smooth lines, results of approximation by Hill equation: Imax ~ 332 ± 44 pA, K_1/2_ ~ 615 ± 77 µM, h ~ 5 ± 1.9 at pH = 8.0 (dark blue, number of cells sensitive to VUAA1—11 out of 17 tested); I_max_ ~ 216 ± 23 pA, K_1/2_ ~ 550 ± 49 µM, h ~ 4 ± 1.4 at pH = 5.5 (light blue, number of cells sensitive to VUAA1—6 out of 9 tested). Current values (*abs(I)*) for analysis in (**C**), (**D**) were obtained at -50 mV. Insets in (**C**), (**D**) show the same data sets where current values were normalized to maximum, individually for every cell tested, and averaged.

To reduce confounding factors and provide better control over experimental conditions, specifically, intracellular pH, we also characterized CpomOrco or CpomOrco^Q417H^ using whole-cell voltage-clamp recordings. HEK cells were co-transfected with CpomOrco or CpomOrco^Q417H^ and GFP. Only GFP positive cells were used in the experiments to ensure expression of the receptors. The cells were patch-clamped and dialyzed with intracellular solutions of different pH (pH 5.5 or 8.0) through recording pipettes. After establishing whole-cell configuration, the cells were activated by different concentrations of VUAA1, dose–response characteristics were generated for different pH conditions and compared (Fig. 5a,b). Consistent with the calcium imaging experiments, the pH sensitivity of CpomOrco was more pronounced than the pH sensitivity, if any, of CpomOrco^Q417H^ (Fig. 5c,d). Low pH reduced the amplitude of responses to saturating agonist concentration (1000 µM VUAA1) from ~ 784 to ~ 196 pA and shifted CpomOrco sensitivity to agonist to EC50 ~ 561 µM versus EC50 ~ 403 µM at pH = 8.0. In contrast, CpomOrco^Q417H^ appeared to be less susceptible to intracellular acidification: Imax ~ 332 pA (pH_i_ = 8.0) versus 216 pA (pH_i_ = 5.5) and EC50 ~ 550 µM (pH = 5.5) versus ~ 615 µM (pH = 8.0).

## Discussion

This study compares functional properties of Orco from *C. pomonella* with a site-directed mutagenized version of its coding sequence, CpomOrco^Q417H^, in which the ICL-3 glutamine at position 417 was mutagenized to histidine. The mutagenization was performed to simulate the polypeptide sequence, at this position, for most insects (Figs. 1, 2), given the observation of histidine as the most frequent amino acid in this position, with the aim to test a possible influence of specific substitutions on physiological properties of Orco.

Polypeptide sequence alignment and phylogenetic analysis demonstrated occurrence of the ICL-3 histidine (H) residue substituted to glutamine (Q) within Ditrysia, contrary to more ancestral moths provided with an ICL-3 H like most of the insects from different orders (Fig. 1b, Supplementary Dataset File). Furthermore, substitution of the ICL-3 H to Q was also observed among representatives of unrelated insect orders, diverged earlier than Lepidoptera (Fig. 2, Supplementary Fig. S1), including within Hymenoptera, as well as more ancient-divergent insects among Blattodeans and Phtirapterans. Generally, the conservation of the H in the ICL-3 resembles a primary stable condition for Orco, while substitution to other amino acid residues is encountered only among a limited number of insects. Although, within Ditrysia, the conservation of Q rather than H may suggest the assumption of a renewed evolutionary stability for the ICL-3 domain of Orco, as this substitution is conserved for all the representatives of this Neolepidopteran subgroup.

Polypeptide sequence analysis of more primitive insects demonstrated occurrence of different amino acid substitutions of the ICL-3 H, including arginine (R) in *P. siccifolium* and *B. tabaci*, serine (S) in *D. citri* and *P. solenopsis* and proline (P) in *T. domestica*. More data from a wider array of Orcos may be explored by future genomic and transcriptomic analysis to shed light about the possible occurrence and impact of similar substitutions in the Orco polypeptide sequences of other representatives.

Interaction of Orco with OR subunits has been proposed to be mediated by intracellular loops^12,40,54^. Among ICLs, it is generally known that the ICL-3 contains regions of Orco and OR interaction for the formation of heteromeric complexes^13^. More recent studies provide some evidence of interactions of ICL-3 s within Orco+OR heteromers to line the interface of the cation channel pore intracellularly^54^. Indeed, Cryo-EM analysis demonstrated involvement of ICL-3 and part of the ICL-2 in constituting the anchor domain, a three-dimensional composition that occludes the vestibule at the intracellular face of the membrane, presenting a barrier to the flow of ions into the cytosol^6^. Furthermore, 3-D modelling of insect ORs and Orco, based on the identification of pairs of amino acids that are important for protein structure and function, named evolutionary couplings (ECs)^55^, predicted the ICL-3 housing clusters of conserved couplings of residues. The identification of these clusters correlates with strong evolutionary constraints on the ICL-3^56^. As proposed by Hopf et al*.*^55^, the very high frequency of strong evolutionary conserved residues within ICL-3 may be explained by the involvement of this loop in forming at least part of the molecular interface for assembly of Orco and ligand-specific ORs into heteromeric complexes.

Studies on protein–protein interaction show the importance of groups of residues including tryptophan (W), tyrosine (Y), and arginine (R) composing “hot-spots” of binding energy within dimer interfaces^40,49,57^. In a more recent report, Miller and Tu^46^ identified some of these residues within Orco ICL-3 and proposed the existence of conserved motifs as models for protein–protein interaction shared among the highly diverse family of insect odorant receptors. In our polypeptide sequence analysis of the ICL-3 of insect Orcos provided with a substitution of the conserved histidine (Supplementary Fig. S1), we demonstrated that a conservation of all the key-residues of the models proposed by Miller and Tu^46^ persists, except for the *B. tabaci* and *P. solenopsis* Orcos. Interestingly, the ICL-3 histidine is located directly upstream of a conserved tryptophan residue (W). Evidence from Miller and Tu^46^ reported this tryptophan to be among the most conserved amino acid residues within the ICL-3 polypeptide sequence of all insects’ Orco and OR subunits, as a possible candidate to constitute part of a “hot spot” for binding energy in protein–protein interfaces involved in this loop.

Cryo-EM analysis of the Orco tetramer of *Apocrypta bakeri* demonstrated projection of this tryptophan (W419) and a tyrosine (Y415) into a pocket formed by a conserved histidine (H333) and other conserved residues (V330, V336, V433, C434, Q436, C437) of the ICL-2 (S5) and part of the ICL-3 (S7a) of a neighbouring subunit^6^. The role of tryptophan and the conserved residues have been proposed in inter-subunit interfaces to stabilize interactions between Orco subunits. In our polypeptide sequence alignment, these residues are conserved overall (Supplementary Dataset file). Among the few exceptions, a substitution in *D. citri* to serine was identified in the respective position of C434^6^, which is interesting since for this representative we observed a substitution to serine also for the ICL-3 histidine (Figs. 1b, 2, Supplementary Fig. S1), and the hydrophilic nature of a serine within the anchor domain suggests possible influences for Orco/OR interactions. Among other representatives, including some of the subgroup Ditrysia, additional substitutions to isoleucine were identified for the respective positions of V336 (Msex, Hmel, Phum, Btab, Sinv, Amel, Mrot, Ccin) and V433 (Psic, Aqua) (Supplementary Dataset file). Although interesting, isoleucine is among the most common amino acids founds in insect ORs at these positions^6^, and in this case it represents a conserved substitution due to similar biochemical properties with valine.

Localization of the ICL-3 histidine residue directly upstream of the highly conserved tryptophan of the anchor domain suggests a possible implication of this histidine and its respective substitutions in the nature of the Orco protein and, possibly, in the interactions between Orco and OR subunits or between the heteromer and its specific ligands. Indeed, a more recent investigation reported VUAA-binding properties among several mutagenized versions of DmelOrco and it demonstrated enhancement of VUAA-sensitivity and a tenfold decrease in the potency to ligand binding for DmelOrco+OR22a when Orco ICL-3 cysteines were mutagenized to serine, including C429 proximal to the highly conserved tryptophan^37^. Additional studies demonstrated that ICL-3 cysteines have the potential to affect assembling of functional tertiary structures through formation of intracellular disulphide bridges^39^, which may, in certain aspects, explain the enhancement of VUAA-sensitivity verified by Turner et al*.*^37^ for the DmelOrco mutagenization C429S. In the context of decrement of potency for binding the OR22a-ligand, Turner et al.^37^ suggested instead that mutations of cysteines may affect the interaction of Orco with DmelOR22a or the trafficking of heteromeric complexes to the cell membrane. In addition, analysis of three-dimensional structures and functional domains of Orco included C429, as well as other ICL-3 cysteines, among the predicted evolutionary conserved residues of the subunit^55^. This gives further support to the importance of amino acids proximal to the highly conserved tryptophan in Orco-VUAA-ligand binding, OR-odorant ligand binding, and possibly in the Orco/OR subunit interactions.

Our polypeptide sequence alignment of Orco ICL-3 confirmed conservation of the respective cysteine reported by Turner et al*.*^37^, which is the amino acid residue directly upstream from the ICL-3 histidine (Fig. 1b, Supplementary Fig. S1). Although speculative, the findings from Turner et al*.*^37^, Corcoran et al*.*^39^ and Hopf et al.^55^ supporting influence on Orco functional properties by cysteines of the ICL-3, may open consideration of possible interplays mediated by residues proximal to these cysteines. In this scenario, residues of functional importance such as cysteine^37,39,55^, and tryptophan^6,46^ proximal to the ICL-3 histidine of our investigation, may constitute with the histidine a structural/functional “ICL-3 hot spot” involved in ligand binding properties and subunit interaction of Orco.

Based on our findings, functional properties of the Ditrysian polypeptide sequence of CpomOrco seem to be affected by a site-directed reversion to histidine in the ICL-3, in terms of an overall reduced expression level of the functional receptor and reduced sensitivity to the VUAA1 agonist and to the pear ester ligand when cells expressed Orco+OR3 heteromers (Fig. 3). Whether malfunctional expression levels of Orco mutant is potentially determined by its overall expression level or rather its malfunctional assembly, we do not know. Comparing EBFP fluorescence (CMV-promoter functionality) with VUAA1 sensitivity (functional expression) the latter possibility seemed more likely if, in general, EBFP expression is considerably (spatially and quantitatively) overlapped with the response to VUAA1. Although in few cases there is a difference in the percentages between EBFP fluorescence and VUAA1 responsiveness (Supplementary Table S1), in any experiment we conducted the predominant majority of the cells responding to VUAA1 were also EBFP positive. Contrary, CpomOrco^Q417H^ transfection always yielded greatly reduced numbers of VUAA1 sensitive cells as an indication of the effects of this amino acid possibly compromising functional assembly.

Taken together, these results suggest the Ditrysian CpomOrco to be more thoroughly adapted to the presence of the glutamine residue in the ICL-3, as reversion to the otherwise conserved histidine is suboptimal for the function of the transmembrane protein. In addition, these findings provide further support to the documented importance of the conservation of the amino acids residues located within the ICL-3 loop, and they contribute evidence of the possible involvements of this domain and its residues in ligand binding^37^ and protein–protein interaction^13,54,58^. Indeed, despite the renowned importance of ICL-3 to Orco/OR interactions^55^ and plasma membrane targeting^13^, to the best of our knowledge there have been no mutagenization studies indicating possible influences from single amino acid substitutions of ICL-3 in Orco/OR interactions. Rather, these effects also represent a possible proximate cause, in light of the evidences, of the reduced ligand binding associated with the amino acid substitutions in proximity to the conserve tryptophan residue^37,39^. Further studies (e.g. utilizing alternative expression systems including *Drosophila* Orco knock-out lines^2^) may help to unveil a more detailed role of specific amino acid substitutions of the ICL-3 histidine in fine tuning functional properties of Orco proteins as chaperones^2,13,22,23^ and/or ionotropic receptor-channels^6^.

The conjugate acid of the imidazole side chain in histidine has a pKa of approximately 6.0. Protonation of the imidazole side chain of the ICL-3 histidine by intracellular acidification may influence sterical and binding properties of the ICL-3. To test this hypothesis we exposed cells to extracellular pH = 4.7 assuming proportional cytoplasmic acidification (Fig. 4). Indeed, the reduction of BCECF (pH probe) fluorescence intensity during incubation with low pH suggested intracellular acidification, which was only partially reversible. We are aware of potential confounding effects generally associated with acidification of extra/intracellular environment including general protonation of amino acid residues of the Orco protein, overall increase in fluorescence intensity of the Fluo-4 probe, decrease in Fluo-4 calcium binding affinity, as well as calcium binding affinity of multiple endogenous calcium buffering systems^59–61^. Thus, the results of these experiments cannot be deemed to be a definitive argument for possible pH_i_ effects on the receptor function. We therefore used the data for rather relative comparisons of CpomOrco or CpomOrco^Q417H^ activity. Nonetheless, the similarity of the pH effects on the dose–response characteristics obtained by calcium imaging (Fig. 4) and patch-clamp electrophysiology (Fig. 5) is compelling.

Interestingly, the relationship between protons and Orco proteins, especially Ditrysian Orcos, appear to be more intimate than might be expected. Ligand binding to Orco/OR complexes is initiated by Odor/Pheromone Binding Proteins (OBPs/PBPs), which, in most cases, display high affinity for specific ligands at the alkaline pH of the sensillar lymph^58^. Constant pH molecular dynamics and molecular docking computational studies demonstrated that localization of low pH on the surface of OSN dendrites^62–64^ induce protonation of BPs disrupting H-bonds among specific amino acid residues present in the ligand binding sites to lead to conformational variation releasing the bound ligand^65^.

Up to now, there is only indirect evidence suggesting the range of pHs that may facilitate turning OBPs to release of ligands^65–67^. Within Ditrysia, the transition midpoint of *B. mori* BmorPBP1 to the release of the ligand bombycol is initiated at pH = 5.37^68^; in *C. pomonella*, a recent study demonstrated significantly reduced affinity of CpomPBP1 to the main ligand codlemone starting from pH = 5.5^69^; in *Lymantria dispar*, it has been shown that while the PBP-pheromone complexes may dissociate releasing of the pheromone at low pHs (5.0–6.0), the affinity of the PBP for pheromone does not vary significantly at physiologically relevant combinations of KCl concentration and pH^70^. Little is known, instead, about actual proton concentration in the insect sensilla endolymph in general and in the proximity to the OSN membrane in particular. As mentioned above, different pH conditions of the extracellular side of the plasma membrane, beyond physiologically relevant pH range (~ 6.8–7.6), would shift intracellular pH with ∆pH_e_/∆pH_i_ ~ 1^53^. With the lack of evidence of specific pH values proximal to the extracellular side of the plasma membrane of insect OSNs, it is possible to speculate that different pH metrics among different insect orders may counterpart fixation of specific amino acid substitutions within intracellular conserved regions of Orco, like ICL-3. Specific amino acid substitutions may impart different susceptibilities to the intracellular pH variation, as we observed for the ICL-3 glutamine in substitution of histidine in the ICL-3 of a Ditrysian Orco (Fig. 4, 5).

In the *Drosophila* olfactory system, acidic pH conditions are sensed by members of the Ionotropic Receptors (IRs) family, among which, several have been described for binding organic acids^71^, including a sensor for carbon dioxide: IR64a^72^. Indeed, part of the IR64a expressing neurons project to DC4 glomeruli, activated by several acidic odorants and to the sole CO_2_-metabolite carbonic acid. Lack of activation of these IR64a neurons to the other CO_2_-metabolite bicarbonate suggests specificity for this IR as a detector of acidification triggered by increased concentrations of CO_2_. Apart from IR64a, GR21a and GR63a are conserved CO_2_-sensors among insects from different orders^73^, although demonstrations are missing to justify their activation mediated by a direct CO_2_-binding, binding of bicarbonate ions or response to acidification of the sensillar lymph related with the presence of CO_2_ in the environment^74^. In acidic sensing modalities involving chemosensory receptors such as IRs and (maybe) GRs, a specific sensor detects the acid moiety and information is conveyed directly to the brain^71–73^. In a different scenario, effects of reduced current associated to acidic pH conditions are renowned since long ago among cation channels permeable to Calcium of different organisms^75,76^, and more recent evidence re-conducts the same effects also to insects^77^. Contrary to our expectations, protonation of the ICL-3 histidine doesn’t seem to affect ligand-binding characteristics, but we rather demonstrated the case for the wild type CpomOrco with glutamine rather than histidine. In this scenario, possible pH_i_ effects on the receptor function (Figs. 4, 5) may suggest a role for cation channels formed by Orcos not provided with ICL-3 histidine, but rather with glutamine, as in Ditrysia, in overall mechanisms of response to acidic conditions, by reducing amplitude and sensitivity of ligand binding at low pH. To our knowledge, this is the first study reporting direct effects of acidic pH on insect Orco/OR channels, which represented a matter of investigation only among mechanisms of ligand binding-and-release from BPs to ORs^58,62–70^.

In this investigation, we demonstrate evidence of altered functional properties of an Orco, based upon mutagenization of a highly conserved ICL-3 residue, which is located in a loop constituting part of the anchor domain of the protein, and it lies in direct proximity to residues with a documented importance for ligand binding and protein–protein interaction. This mutagenization influences the overall functional expression of the Orco subunit and reduces the sensitivity to ligand, but it preserves VUAA1-sensitivity at extracellular and intracellular acidic conditions. Our data support the idea of the existence of a possible “hot spot” made of three residues of the ICL-3, such as cysteine, histidine and tryptophan (CHW), with a possible involvement in ligand binding properties, subunit interactions and pH susceptibility. Variability of the second position of this candidate “hot spot” was demonstrated among the Neolepidopteran subgroup Ditrysia, based on a conservation of glutamine rather than histidine, which suggests an enhanced evolutionary stability for the ICL-3 domain of Orco. We propose the existence of ICL-3 amino acid substitution providing functional adaptation (tuning) of Ditrysian receptors to extra-/intracellular pH variation. Susceptibility in agonist-binding at acidic pH conditions for the Ditrysian receptor, missing in the histidine-mutagenized version, also suggests potentials of the Orco/OR cation channels for this subgroup of Lepidoptera in some sort of involvement in sensing acidic pH.

To verify our hypothesis, future efforts may address the isolation and the functional characterization of novel Orco coding sequences among Ditrysian representatives and as well as among insects from different orders provided with alternative substitutions from histidine in the ICL-3.

## Methods

### Sequence alignment of insect olfactory co-receptors and bioinformatics

The polypeptide sequence of CpomOrco (AFC91712.1) was used as query in search of deposited sequences in protein BLAST (https://blast.ncbi.nlm.nih.gov/Blast.cgi), to identify polypeptide sequences from some of the most representative species among different insect orders, which were manually aligned using BioEdit v7.2.5^78^. In the dataset (Supplementary Dataset File), sequences are reported by use of acronyms listed in Table 1. The polypeptide sequences of *Lampronia capitella* and *Rhyacophila nubila* Orco were kindly provided by Dr. Jothi Kumar Yuvaraj. The polypeptide sequence of *Synanthedon myopaeformis* Orco was kindly provided by Dr. William B Walker III. The polypeptide sequence of *Calyptra thalictri* Orco was kindly provided by Dr. Sharon Hill.

**Table 1:** Acronyms of the Orco polypeptide sequences.

*Species name*	Author	Order	Family	Accession number	Source	Acronym
*Cydia pomonella*	L	Lepidoptera	Tortricidae	AFC91712.1	GenBank	Cpom
*Epiphyas postvittana*	Walker	Lepidoptera	Tortricidae	ACJ12928.2	GenBank	Epos
*Spodoptera littoralis*	Boisduval	Lepidoptera	Noctuidae	ABQ82137.1	GenBank	Slit
*Calyptra thalictri*	Borkhausen	Lepidoptera	Noctuidae	–	Sharon Hill	Ctha
*Synanthedon myopaeformis*	Borkhausen	Lepidoptera	Sesiidae	–	William B. Walker III	Smyo
*Dendrolimus kikuchii*	Matsumura	Lepidoptera	Lasiocampidae	AII01079.1	GenBank	Dkik
*Antheraea pernyi*	Guérin-Méneville	Lepidoptera	Saturnidae	AJ555486.1	GenBank	Aper
*Manduca sexta*	L	Lepidoptera	Sphingidae	ACM18060.1	GenBank	Msex
*Bombyx mori*	L	Lepidoptera	Bombycidae	NP_001037060.1	GenBank	Bmor
*Lymantria dispar asiatica*	Vnukovskij	Lepidoptera	Erebidae	AHA50097.1	GenBank	Ldis
*Conogethes punctiferalis*	Guenée	Lepidoptera	Crambidae	AGF29886.1	GenBank	Cpun
*Amyelois transitella*	Walker	Lepidoptera	Pyralidae	NP_001299600.1	GenBank	Atra
*Plutella xylostella*	L	Lepidoptera	Plutellidae	BAG71421.2	GenBank	Pxyl
*Heliconius melpomene*	L	Lepidoptera	Nymphalidae	AQQ73487.1	GenBank	Hmel
*Argyresthia conjugella*	Zeller	Lepidoptera	Yponomeutidae	AEA76288.1	GenBank	Acon
*Papilio machaon*	L	Lepidoptera	Papilionidae	XP_014363049.1	GenBank	Pmach
*Eriocrania semipurpurella*	Stephens	Lepidoptera	Eriocraniidae	ATV96621.1	GenBank	Esem
*Lampronia capitella*	Clerck	Lepidoptera	Prodoxidae	–	Jothi Kumar Yuvaraj	Lcap
*Zootermopsis nevadensis*	Hagen	Blattodea	Thermopsidae	KDR12002.1	GenBank	Znev
*Cryptotermes secundus*	Hill	Blattodea	Kalotermitidae	XP_023716643.1	GenBank	Csec
*Pediculus humanus corporis*	L	Phtiraptera	Pediculidae	EEB12924.1	GenBank	Phum
*Thermobia domestica*	Packard	Zygentoma	Lepismatidae	–	^24^	Tdom
*Psyllum siccifolium*	L	Phasmatodea	Phyllidae	–	^24^	Psic
*Rhyacophila nubila*	Zetterstedt	Trichoptera	Rhyacophilidae	–	Jothi Kumar Yuvaraj	Rnub
*Apolygus lucorum*	Meyer-Dür	Hemiptera	Miridae	AHC72290.1	GenBank	Aluc
*Cimex lectularius*	L	Hemiptera	Cimicidae	NP_001303637.1	GenBank	Clec
*Acyrthosiphon pisum*	Harris	Hemiptera	Aphididae	XP_001951646.2	GenBank	Apis
*Phenacoccus solenopsis*	Tinsley	Hemiptera	Pseudococcidae	ANW12106.1	GenBank	Psol
*Bemisia tabaci*	Gennadius	Hemiptera	Aleyrodidae	XP_018916513.1	GenBank	Btab
*Diuraphis noxia*	Börner	Hemiptera	Aphididae	XP_015371514.1	GenBank	Dnox
*Diaphorina citri*	Kuwayama	Hemiptera	Psylloidea	XP_008484015.1	GenBank	Dcit
*Schistocerca gregaria*	Forsskål	Orthoptera	Acrididae	AEX28371.1	GenBank	Sgre,
*Drosophila melanogaster*	Meigen	Diptera	Drosophilidae	Q9VNB5.2	GenBank	Dmel
*Drosophila suzukii*	Matsumura	Diptera	Drosophilidae	XP_016931849.1	GenBank	Dsuz
*Musca domestica*	L	Diptera	Muscidae	AFH96944.1	GenBank	Mdom
*Ceratitis capitata*	Wiedemann	Diptera	Tephritidae	AAX14775.1	GenBank	Ccap
*Calliphora stygia*	Fabricius	Diptera	Calliphoridae	AID61201.1	GenBank	Csty
*Anopheles gambiae*	Giles	Diptera	Culicidae	Q7QCC7.3	GenBank	Agam
*Culex quinquefasciatus*	Say	Diptera	Culicidae	ABB29301.1	GenBank	Cqui
*Holotrichia oblita*	Faldermann	Coleoptera	Melolonthidae	AEE69033.1	GenBank	Hobl
*Ambrostoma quadriimpressum*	Schönherr	Coleoptera	Chrysomelidae	AJF94638.2	GenBank	Aqua
*Dendroctonus ponderosae*	Hopkins	Coleoptera	Curcolionidae	XP_019768125.1	GenBank	Dpon
*Tribolium castaneum*	Herbst	Coleoptera	Tenebrionidae	EFA05687.1	GenBank	Tcas
*Solenopsis invicta*	Buren	Hymenoptera	Formicidae	XP_011164243.1	GenBank	Sinv
*Apis mellifera*	L	Hymenoptera	Apidae	NP_001128415.1	GenBank	Amel
*Megachile rotundata*	Fabricius	Hymenoptera	Megachilidae	XP_012146523.1	GenBank	Mrot
*Chouioia cunea*	Yang	Hymenoptera	Eulophidae	AIY24336.1	GenBank	Ccun
*Ceratosolen solmsi marchali*	Mayr	Hymenoptera	Agaonidae	NP_001292395.1	GenBank	Csol
*Nasonia vitripennis*	Walker	Hymenoptera	Pteromanidae	NP_001164465.1	GenBank	Nvit
*Cephus cinctus*	Norton	Hymenoptera	Cephidae	NP_001310774.1	GenBank	Ccin
*Macrocentrus cingulum*	Brischke	Hymenoptera	Braconidae	AGI62937.1	GenBank	Mcin
*Neodiprion lecontei*	Fitch	Hymenoptera	Diprionidae	XP_015513389.1	GenBank	Nlec

Acronyms are reported in the analysed dataset (Supplementary dataset file), among figures and in the text. Accession number of polypeptide sequences and sources are indicated.

To generate topological predictions (Fig. 1), polypeptide sequences were submitted on TOPO 2.0 (http://www.sacs.ucsf.edu/cgi-bin/open-topo2.py) integrating transmembrane predictions from TOPCONS (http://topcons.cbr.su.se). Outputs were edited with Adobe Illustrator. Orco ICL-3 of insect representatives provided with an amino acid substitution at the respective position with the 417th residue of the amino acid sequence of CpomOrco were compared by polypeptide sequence alignment. In an analysis of the most critical amino acid residues with possible involvement in protein–protein interactions (Supplementary Fig. S1), we compared motif A-predictions for *An. gambiae*, *D. melanogaster* and *A. mellifera* models reported by Miller and Tu^46^.

### Phylogenetic analysis

Amino acid sequences of Orcos were aligned using MAFFT online version 7.220 (http://mafft.cbrc.jp/alignment/server/phylogeny.html) through the FFT-NS-i iterative refinement method, with JTT200 scoring matrix, unalignlevel 0.3, “leave gappy regions” set, and other default parameters^79^. Aligned sequences were used to calculate the evolutionary history of receptors of each gene family with MEGA7 software^80^ in command line, with the following parameters: Maximum Likelihood Tree Method with the JTT-F’ model, uniform rates, use all sites, nearest neighbor interchange heuristic method, very strong branch swap filter and default automatic NJ/BioNJ initial tree. The bootstrap consensus of each phylogenetic tree was inferred from 600 replicates. Output consensus Newick format trees were compiled with MEGA5 software^81^ and edited with Adobe Illustrator. Nomenclature was adapted based on Table 1.

### Site-directed mutagenesis of CpomOrco

To generate a mutagenized version of CpomOrco, *Donr_Orco_Nt* (5′-*attB*1-cacc-ATGATGGGTAAAGTGAAATCTCA-3′) and *Donr_Orco_Ct* (5′-attB2-TTACTTCAGTTGTACTAACACCATGA) primers have been designed on the start and the stop codons of the original coding sequence^82^, providing additional *attB* regions (*attB1* forward region: 5′-GGGGACAAGTTTGTACAAAAAAGCAGGCTTAACA-3′; *attB2* reverse region: 5′GGGGACCACTTTGTACAAGAAAGCTGGGT-3′) for Gateway Technology recombination (Invitrogen, Life technologies, Waltham, MA, USA). Amplification with these primers was conducted coupling primers specific for site-directed mutagenesis (*Cpom_SDM_Rv*: 5′-GTCGTACCAGTGGCAGGAGTA-3′, Tm = 63.2 °C and *Cpom_SDM_Fw*: 5′-TACTCCTGCCACTGGTACGAC-3′, Tm = 63.2 °C). In particular, mutagenizing primers were designed to overlap the nucleotide position 1251 including a cytosine in substitution of an adenine on the sense strand (*Cpom_SDM_Fw*: TACTCCTGCCACTGGTACGAC, Tm = 63.2 °C) and a guanine in substitution of a thymine on the antisense strand (*Cpom_SDM_Rv*: GTCGTACCAGTGGCAGGAGTA, Tm = 63.2 °C). To extend the N-terminus fragment (1302 bp), amplification was performed combining the N-terminal forward primer with *Cpom_SDM_Rv* primer. To extend the C-terminus fragment (227 bp), amplification was performed combining the C-terminal reverse primer with *Cpom_SDM_Fw* primer. Amplification was conducted with thermostable DNA polymerase adopting a temperature program of 94 °C for 5 min, followed by 40 cycles of 94 °C for 1 min, Tm of the *Donr* primer for 1 min, 72 °C for 1 min and 15 s, and a final elongation of 72 °C for 7 min. PCR products were analyzed by electrophoresis on a 1.5% agarose gel, stained with ethidium bromide and visualized using a Gel Doc XR (Bio-Rad, Hercules, CA, USA). Amplicon-bands were excised and purified using the QIAquick Gel extraction kit (Qiagen, Hilden, Germany) and quantifications of 144.4 ng/µL and 18.91 ng/µL was estimated using Nanodrop (Nanodrop 8000 UV–vis Spectrophotometer, Thermo Scientific, Wilmington, DE, USA).

Volumes of purified amplicons were combined based on their bp-lengths and concentration in order to start fusion-PCR amplification with the same amount of DNA-fragments in the final template. In brief, 1.0 µL of N-terminus (144.4 ng) was mixed with 1.33 µL of C-terminus (25.18 ng) to have a nanogram-ratio (Ct_ng_/Nt_ng_ = 0.174) identical with their bp-length-ratio (Ct_lenght_/Nt_Lenght_ = 0.174). Volumes were mixed with 12.5 µL of GoTaq Green Master Mix (Promega, Fitchburg, WI) and brought to a final volume of 25 µL with water. An initial treatment of denaturation and extension of amplicons was conducted with a temperature program of 94 °C for 5 min and 72 °C for 5 min. After extension, primer aliquots of 0.5 µL *Donr_Orco_Nt* (attB1-cacc-ATGATGGGTAAAGTGAAATCTCA, Tm = 57.6 °C) and 0.5 µL *Donr_Orco_Ct* (attB2-TTACTTCAGTTGTACTAACACCATGA, Tm = 61.7 °C) were added to the reaction volume to undertake an additional amplification using a temperature program of 94 °C for 5 min, followed by 40 cycles of 94 °C for 1 min, 57.6 °C for 1 min, 72 °C for 2 min, and a final elongation of 72 °C for 7 min. The reaction volume was analysed on gel electrophoresis validating a band at approximately 1500 bp. The sequence of the mutagenized Orco (*CpomOrco*^*Q417H*^) was confirmed by Sanger (Sanger sequencer, 3730xl Applied Biosystems, Life Technologies) after gel extraction and quantification.

### Cloning of the mutagenized CpomOrco^***Q417H***^

The mutagenized coding sequence (CDS) of CpomOrco^Q417H^ was cloned using the same procedures we previously described^14^. A 4.0 µL aliquot of PCR volume was mixed with 1.0 µL of BP-clonase (Gateway Technology, Invitrogen) and 150 ng of pDONR221 (Invitrogen), and was incubated for 4 h at 25 °C. Of this reaction volume, 2.0 µL was used to transform TOP10 competent cells (Invitrogen). After transformation, 50 µL of the reaction was plated on 50 µg/mL Kanamycin selective media and incubated overnight at 37 °C. Colonies were sampled, and diluted in 50 µL selective LB media with 50 µg/mL kanamycin, to be grown for 2.0 h at 37 °C and 225 rpm. Colony PCR was performed to confirm inserts, using 1.0 µL culture from single colony-volumes with the M13FW universal primer and *Donr_Orco_Ct* primer. Amplifications were performed using the GoTaq Green Master Mix (Promega, Fitchburg, WI, USA) with a temperature program of 95 °C for 15 min, followed by 35 cycles of 95 °C for 45 s, 55 °C for 1 min, 72 °C for 2 min, and a final elongation of 72 °C for 7 min. Colony PCR samples were analysed by electrophoresis on 1.5% agarose gel. Bands were visualized after staining with ethidium bromide using a Gel Doc XR (Bio-Rad). Cultures producing relevant bands in colony PCR were grown at 37 °C and 225 rpm overnight in 5.0 mL selective LB media with 50 µg/mL kanamycin. The pDONR221 plasmids containing *CpomOrco*^*Q417H*^ ORF were purified using a miniprep kit (Qiagen). Plasmid quantification was performed using Nanodrop (8000 UV–vis Spectrophotometer), samples were sequenced (Sanger sequencer, 3730xl) using M13 universal primers. To transfer *CpomOrco*^*Q417H*^ ORF to a destination vector for HEK293A heterologous expression, 100 ng pDONR221-*CpomOrco*^*Q417H*^ was mixed with 150 ng pcDNA40-DEST (Invitrogen), 2.0 µL LR-clonase (Invitrogen) and TE-buffer to a final volume of 10 µL and incubated overnight at 25 °C. After incubation, 1.0 µL Proteinase K was added to interrupt the reaction and the volume was incubated at 37 °C for 10 min. A 1.0 µL final reaction volume was used to transform TOP10 competent cells (Invitrogen). After transformation, 50 µL of the reaction was plated on 100 µg/mL ampicillin selective media and incubated overnight at 37 °C. In search for positive colonies, colony PCR was performed as described above using 100 µg/mL ampicillin for selection and amplifying samples with *Donr-*primers. Colony PCR samples were analysed by gel electrophoresis and cultures producing relevant bands in colony PCR were grown at 37 °C and 225 rpm overnight in 5.0 mL selective LB media with 100 µg/mL Ampicillin, to purify pcDNA40-DEST plasmid containing *CpomOrco*^*Q417H*^ CDS. The plasmid was quantified by Nanodrop, sequence was confirmed by Sanger using universal primers (CMV: 5′-CGCAAATGGGCGGTAGGCGTG-3′, BGH 5′-TAGAAGGCACAGTCGAGG-3′), *Donr_Orco_Nt* and *Donr_Orco_Ct* primers.

### Heterologous expression in HEK293 cells and transient transfection

HEK293 cells lines (HEK293A/HEK293T) were grown in HEK cell media [Dulbecco's modified Eagle's medium containing 10% fetal bovine serum (MP Biomedicals, Solon, OH, USA), 2.0 mM L-glutamine, and 100 µg/mL penicillin/streptomycin (Invitrogen)] at 37 °C and 5% CO_2_. To test transient expression of wild type *CpomOrco* and *CpomOrco*^*Q417H*^ variant for calcium imaging and patch-clamp recording, 35-mm petri dishes containing semi-confluent HEK293A cells were transiently transfected. Cells were transfected with 0.6 µg of pcDNA40-DEST carrying the coding sequence of the CpomOrco variant (depending on the experiments). To report expression for calcium imaging experiments, 0.6 µg of a separate plasmid DNA [pEBFP2-Nuc, a gift from Robert Campbell (Addgene plasmid # 1489352)] carrying the coding sequence for a blue fluorescent protein (EBFP) was co-transfected. To report expression in patch-clamp recordings, 1.0 µg of a separate plasmid DNA (pXOOM, Clontech, Mountain View, CA, USA) carrying the coding sequence for a green fluorescent protein (GFP) was co-transfected. Expressions of fluorescent reporter genes were under the regulation of the same promoter for Cpom-genes (CMV). For specific experiments, co-transfections were conducted combining an additional 1.2 µg of pcDNA40-DEST/CpomOR3 or pcDNA40-DEST/CpomOR6a, as we previously described^14^. Co-transfections with CpomOR3 were performed for dose–response experiments to pear ester: CpomOR3 was co-expressed with CpomOrco variants by the use of HEK293T (Fig. 3b); given the reported functional co-expression data of the CpomOrco + OR3 heteromers by the use of these specific cells^14^. In brief, transfection DNAs were dissolved in 100 µL sterile DMEM, mixed with 3.0 µL Calfectin (SignaGen, Rockville, MD, USA) following the recommended protocol. Transfections were conducted overnight for up to 18 h, HEK cell media was replaced with 2.0 mL fresh media to incubate cells at 37 °C for up to 6–8 h, at which point part of the cell culture was spread in the middle of a 35-mm plate as individual cells or small clusters and rinsed at the sides with 2.0 mL fresh HEK media. After splitting, cells were allowed to recover for at least 1 day prior to calcium imaging.

### Imaging experiments

Activation of HEK293 cells transfected with *CpomOrco* variants was tested using the same procedures we previously described^14^. Petri dishes were incubated for 1 h at room temperature in 1.0 mL HEK Ca^++^ Ringer (mM: 140 NaCl, 5.0 KCl, 2.0 CaCl_2_, 10 HEPES, pH 7.4) containing the fluorescent calcium indicator Fluo-4AM (Invitrogen) at 5–15 µM prepared with 0.2–0.06% Pluronic F-127 (Invitrogen). To monitor intracellular pH, HEK293A cells were loaded with 10.0 µL BCECF dye (Anaspec, Fremont, CA).

As reported in Cattaneo et al*.*^14^, the buffer was removed after incubation, cells were rinsed with 4.0 mL fresh HEK Ca^++^ Ringer and placed on the stage of an inverted microscope (Olympus IX-71, Olympus Corp., Tokyo, Japan) equipped with a cooled CCD camera (ORCA R2, Hamamatsu, Hamamatsu City, Japan). Cells were continuously superfused with Ca^++^ Ringer using two gravity fed perfusion contours. The stimulating contour washing the cells (~ 250 µL/min) was switched rapidly to the stimulus contour using a multi-channel rapid solution changer (RSC-160, Bio-Logic, Claix, France) under the software control of Clampex 9 (Molecular Devices, Sunnyvale, CA, USA).

Fluorescence imaging was performed using settings optimized for Imaging Workbench 6 software (INDEC BioSystems, Santa Clara, CA, USA)^14^. The non-responsive cells were not included in these analyses. Each cell was assigned a region of interest (ROI) and changes in fluorescence intensity within each ROI were measured and expressed as the fractional change in fluorescence intensity (dF). Stored time series image stacks were analysed off-line using Imaging Workbench 6, Clampfit 10.5 (Molecular Devices) and SigmaPlot 11 (Systat Software Inc., San Jose, CA, USA). Dose–response curves were approximated using Hill equation. Constraints were applied in some cases to fit either limited or greatly scattered datasets. Continuous traces of multiple responses were compensated for slow drift of the baseline fluorescence when necessary. All recordings were performed at room temperature (22–25 °C).

Among the ligands we previously reported active on CpomOrco/OR channels^14^, the non-specific Orco-agonist VUAA1: acetamide,N-(4-ethylphenyl)-2-[[4-ethyl-5-(3-pyridinyl)-4H-1,2,4-triazol-3-yl]thio]-, CAS 525582–84-7 (Glixx Laboratories, Southborough, MA, USA); and the CpomOR3 agonist pear ester: ethyl (E,Z)-2,4 decadienoate, CAS 3025–30-7 (Sigma Aldrich, St. Louis, MO, USA) were dissolved in dimethyl sulfoxide (DMSO, Sigma Aldrich) and stored as a stock solutions (200 mM) at 4 °C. Final working concentrations of VUAA1 and pear ester were prepared right before the experiments. Amplitudes of the calcium responses were used to generate dose–response characteristics. Values were normalized to the response amplitude recorded at the highest concentration.

### Electrophysiological experiments

Electrophysiological experiments were performed according with the same procedures we previously described^14^. Patch pipettes were fabricated from borosilicate capillary glass (BF150-86–10, Sutter Instrument, CA, USA) using a Flaming-Brown micropipette puller (P-87, Sutter Instrument). Only patches with initial cell-attached seal resistance estimated higher than 1.0 GOhms were used in the experiments. Intracellular (pipette) solution for whole-cell experiments was KCl 140 mM, EGTA 1 mM, Hepes 5 mM, MES 5 mM, pH 8.0 or pH 5.5 (depending on the experiment) adjusted with either NaOH or HCl, standard Na^+^ 140 mM. bath solution was usually NaCl 140 mM, EGTA 0.1 mM, Hepes 10 mM, pH 7.4 (adjusted with Tris-base or NaOH). VUAA1 doses (0–1000 µM) were added to the extracellular test solutions.

As described in methods of Cattaneo et al*.*^14^, GFP positive HEK293A cells were visualized using either an Axiovert 100 inverted microscope (Carl Zeiss, Inc., München, Germany) equipped with a mercury vapour compressed-arc lamp (HBO100) coupled to widefield fluorescence filter set (1114–459, Carl Zeiss, Inc.) or Olympus IX-71 inverted microscope described above (Olympus Corp.). The Orco channel-mediated currents were investigated using whole-cell patch-clamp recordings. The currents were measured with a 200B patch-clamp amplifier (Molecular Devices) and a digital interface (Digidata 1320A, Molecular Devices), low pass filtered at 5.0 kHz, sampled at 1–2 kHz. Analysis of the data was carried out using pCLAMP 9.2/10.5 software (Molecular Devices) and SigmaPlot 11 (Systat Software Inc). In some cases, the Orco channel whole-cell current–voltage characteristics were obtained using voltage ramp protocol: series of 15-ms steps at − 100 mV followed by a 150-ms voltage ramps from − 100 mV to + 100 mV applied from a holding potential of − 50 mV. The interval between sweep starts was 1 s. Data were analysed using Clampfit 10.5 (Molecular Devices) and SigmaPlot 11–14 (Systat Software Inc). The non-responsive cells were not included in these analyses.

### Ethical approval

The authors declare that all compounds used in this study were pure according to respective supplier’s standards or sampled from pure stocks used in previously published methods.

## Supplementary Information

Supplementary Information.

## Data Availability

The data that support the findings of this study are available within the article and the associated Supplementary material. Any other data are available from the corresponding author upon request. Correspondence and material request should be addressed to Dr. Alberto Maria Cattaneo, albertomaria.cattaneo@slu.se.
