# Supplementary material for: Altered functional properties of the codling moth Orco mutagenized in the intracellular loop-3
Source: Sci Rep. 2021 Feb 16;11:3893. doi: 10.1038/s41598-021-83024-3 (PMC7887336; doi:10.1038/s41598-021-83024-3)
Supplement: Supplementary file 1 — Supplementary Information. [file 41598_2021_83024_MOESM1_ESM.pdf]

Altered functional properties of the Codling Moth Orco mutagenized in the intracellular loop-3

Yuriy V. Bobkov, William B. Walker III and Alberto Maria Cattaneo*

Supplementary Dataset File. Alignment of the polypeptide sequences of 52 insect odorant receptor co-receptors. Asterisks, residues involved in a possible hydrophobic network with the ICL-3 histidine/glutamine as shown in Butterwick et al.[6]; blue, residues that are conserved in the whole alignment; red, residues which may not be conserved. Note: for *Diaphorina citri* and *Diuraphis noxia* Orcos, most of the polypeptide sequence at the N-terminal is missing: no conclusions can be drawn for the conservation of V330, H333 and V336 residues.

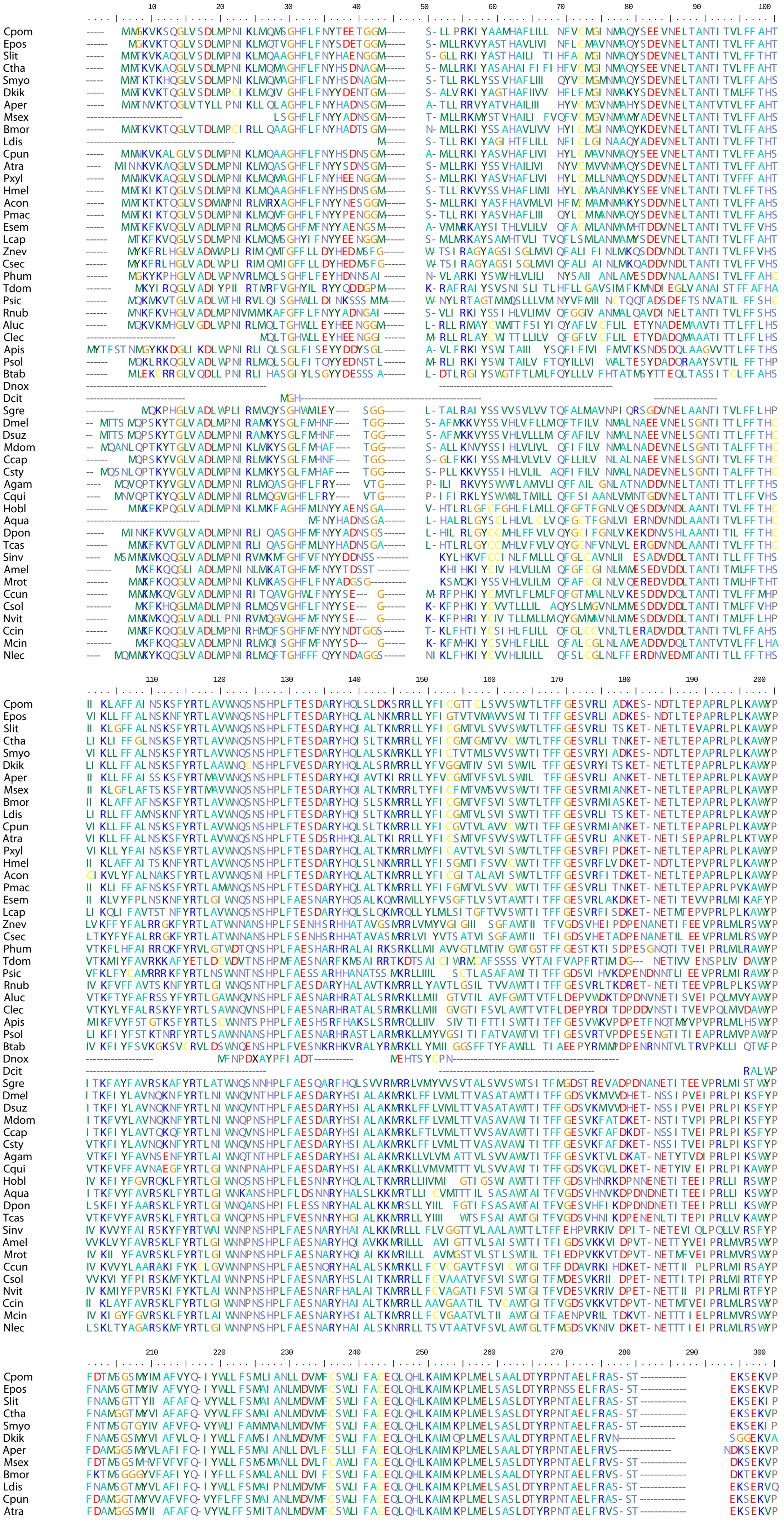

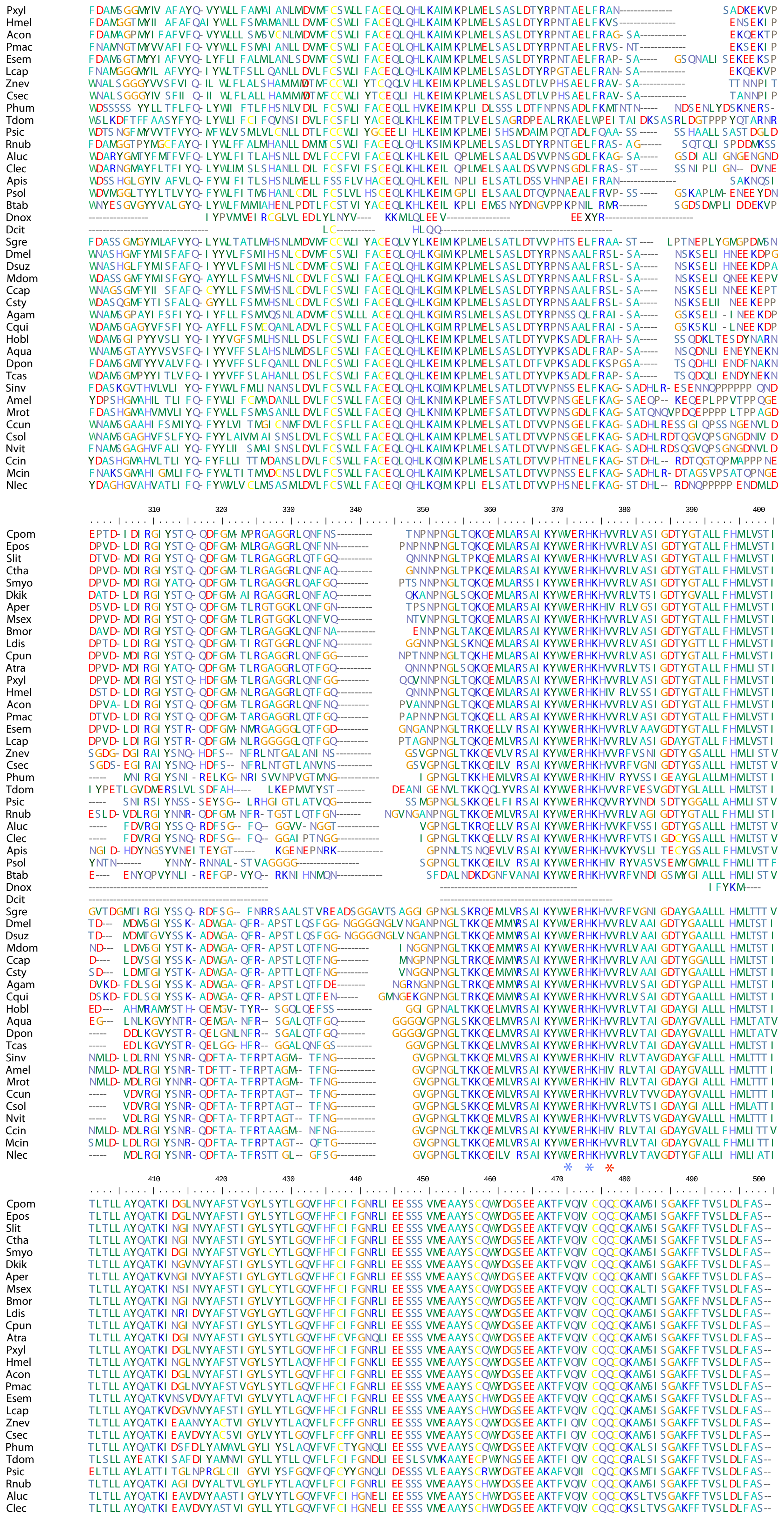

```
Apis    IL TIL AYQATKI NGVNVFAFSTI GYLMYSFAQI FMFCI HGNELI EESSS VMEAAYGCHWYDGSEE AKTFVQIV CQQCQKPLIV SGAKFFNVSLDLFAS--
Psol    TLTLL AYQATKI DGVNVYAFSTI GYLVYTLGQI YTFCI YGNELI DESSS VMEAAYSCSWYDGSEE AKTFVQIV CQQCQKALSVTGAKFFTVSLDLFAS--
Btab    TLTLL AYQATQI TGI NVFGMSVI GYLGYSFI QVYAFCI YGNELI EESSS VMEAAYDCRWYDGSEE AKTFVQIV CQQCQKSLSI SGAKFFTVSLDLFAS--
Dnox    -----------      I NGVNVFAFSTI GYLMYSFAQI FMFCI HGNELI EESSS VMEAAYGCHWYDGSEE AKTFVQIV CQQCQKPLIV SGAKFFNVSLDLFAS--
Dcit    -------     PPTMIEGVNVYAFSTI GYLVYALAQVFFF CFYGNELI EQSSS VMEAAYSCSWYDGSEDAKTFVQIV SQQCQKSLSI TGSKFFTVSLDLFASSF
Sgre    TLTLL AYQATKI DSVDVYAASVLGYLFYTLGQVFLFCVFGNRLI EESSS VMEAAYSCHWYDGSEE AKTFVQIV CQQCQ---------------------
Dmel    KLTLL AYQATKI NGVNVYAFTVVGYLGYALAQVFHFCI FGNRLI EESSS VMEAAYSCHWYDGSEE AKTFVQIV CQQCQKAMSI SGAKFFTVSLDLFAS--
Dsuz    KLTLL AYQATKI NGVNVYAFTVVGYLGYALAQVFHFCI FGNRLI EESSS VMEAAYSCHWYDGSEE AKTFVQIV CQQCQKAMSI SGAKFFTVSLDLFAS--
Mdom    KLTLL AYQATKI TGVNVYAFTVI GYLGYALAQVFHFCI FGNRLI EESSS VMEAAYSCHWYDGSEE AKTFVQIV CQQCQKAMSI SGAKFFTVSLDLFAS--
Ccap    MLTLL AYQATKI TGVNVYAFTTVGYLCYALAQVFHFCI FGNRLI EESSS VMEAAYSCHWYDGSEE AKTFVQIV CQQCQKAMSI SGAKFFTVSLDLFAS--
Csty    KLTLL AYQATKI TGVNVYAFTVI GYLGYALAQVFHFCI FGNRLI EESSS VMEAAYSCHWYDGSEE AKTFVQIV CQQCQKAMSI SGAKFFTVSLDLFAS--
Agam    KLTLL AYQATKI DGVNVYGLTVI GYLCYALAQVFLFCI FGNRLI EESSS VMEAAYSCHWYDGSEE AKTFVQIV CQQCQKAMTI SGAKFFTVSLDLFAS--
Cqui    KLTLL AYQATKI DGLNVYGLTVI GYLVYALAQVFLFCI FGNRLI EESSS VMEAAYSCHWYDGSEE AKTFVQIV CQQCQKAMTI SGAKFFTVSLDLFAS--
Hobl    TLTLL AYQATKI DGVNKYALTVLGYLFYALAQVFHFCI FGNRLI EESSS VMEAAYSCHWYDGSEE VKTFVQIV CQQCQKAMSI SGAKFFTI SLDLFAS--
Aqua    MLTLL AYQATKI DGVNKYAATVI GYLVYSLAQVFHFCI FGNRLI EESSS VMEAAYSCHWYDGSEE AKTFAQII  CQQCQKALSI SGAKFFTI SLDLFAS--
Dpon    MLTLL AYEATKI DGLNTYAATTLGYLLYSLAQVFHFCI FGNRLI EESSS VMEAAYSCHWYDGSEE AKTFVQIV CQQCQKSLFI SGAKFFTI SLDLFAS--
Tcas    MLTLL AYQATKI TGVDKYAATVLGYLLFALAQVFHFCI FGNRLI EESSS VMEAAYSCHWYDGSEE AKTFVQIV CQQCQKAMSI SGAKFFTI SLDLFAS--
Sinv    TLTLL AYQATKVNGI NVYAASTI GYVLYTFGQVFLFCI FGNRLI EESTS VMEAAYSCHWYDGSEE AKTFVQIV CQQCQKAMSI SGAKFFTVSLDLFAS--
Amel    TLTLL AYQATKI HAVDTYAASVVGYLLYSLGQVFMLCI FGNRLI EESSS VMEAAYSCHWYDGSEE AKTFVQIV CQQCQKAMSI SGAKFFTVSLDLFAS--
Mrot    TLTLL AYQATKI HGVDTYSASVI GYLLYSLGQVFMLCI FGNRLI EESSS VMEAAYSCHWYDGSEE AKTFVQIV CQQCQKAMSI SGAKFFTVSLDLFAS--
Ccun    TLTLL AYQATKVNGVNVYAATTI GYLLYTLGQVFLFCVFGNRLI EESSS VMEAAYSCHWYDGSEE AKTFVQIV CQQCQKAMSI SGAKFFTVSLDLFAS--
Csol    TLTLL AYQATKVNGVNVYAATVVGYLLYTLGQVFLFCI FGNRLI EESSS VMEAAYSCHWYDGSEE AKTFVQIV CQQCQKAMSI SGAKFFTVSLDLFAS--
Nvit    TLTLL AYQATKVNGVNVYAATVI GYLLYTLGQVFLFCI FGNRLI EESSS VMEAAYSCHWYDGSEE AKTFVQIV CQQCQKAMSI SGAKFFTVSLDLFAS--
Ccin    SLTLL AYQATKVNTVDVYAATVI GYVLYTLGQVFLFCI FGNRLI EESSS VMEAAYSCHWYDGSEE AKTFVQIV CQQCQKAMSI SGAKFFTVSLDLFAS--
Mcin    SLTLL AYQATKVNGLNVYAATTI GYFSYALAQVFLFCI FGNRLI EESSS VMEAAYSCHWYDGSEE AKTFVQIV CQQCQKAMSI SGAKFFTVSLDLFAS--
Nlec    TLTLL AYQATKI SGFDVYSMGVI GYIL YSLGQVFLFCI FGNRLI EESSS VMDAAYSCQWYDGSEE AKTFVQIV CQQCQKAMSVSGAKFFTVSLDLFAS--
                                                                           *                  * *   **

                   510        520        530
        ....|....|....|....|....|....|....|.

Cpom    -- VLGAVVTYFMVLVQLK------------------
Epos    -- VLGAVVTYFMVLVQLK------------------
Slit    -- VLGAVVTYFMVLVQLK------------------
Ctha    -- VLGAVVTYFMVLVQLK------------------
Smyo    -- VLGAVVTYFMVLVQLK------------------
Dkik    -- VLGAVVTYFMVLVQLK------------------
Aper    -- VLGAVVTYFMVLVQLK------------------
Msex    -- VLGAVV----------------------------
Bmor    -- VLGAVVTYFMVLI QLK------------------
Ldis    -- VLGAVVTYFMVLVQLK------------------
Cpun    -- VLGAVVTYFMVLVQLK------------------
Atra    -- VLGAVVTYFMVLVQLK------------------
Pxyl    -- VLGAVVTYFMVLVQLK------------------
Hmel    -- VLGAVVTYFMVLVQLK------------------
Acon    -- VLGAVVTYFMVLVQLK------------------
Pmac    -- VLGAVVTYFMVLVQLK------------------
Esem    -- VLGAVVTYFMVLVQLK------------------
Lcap    -- VLGAVVTYFMVLVQLK------------------
Znev    -- VLGAVVTYFMVLVQLN------------------
Csec    -- VLGAVVTYFMVLVQLN------------------
Phum    -- VLGAVVTYFMVLVQLK------------------
Tdom    -- VLGAVVTYFMVLI QLK------------------
Psic    -- IL GAVVTYFLLLI  QLN------------------
Rnub    -- VLGAVVTYFMVLVQLK------------------
Aluc    -- VFGAVVTYFMVLVQLK------------------
Clec    -- VLGAVVTYFMVLVQLK------------------
Apis    -- VLGAVVTYFMVLVQLK------------------
Psol    -- VLGAVVTYFMVLI QLN------------------
Btab    -- VLGAVVTYFMVLVQLN------------------
Dnox    -- VLGAVVTYFMVLVQLK------------------
Dcit    VNLLDEYFFFLSVIV DLDDLVCTAFLGYVQSVQTTS
Sgre    -----------------------------------
Dmel    -- VLGAVVTYFMVLVQLK------------------
Dsuz    -- VLGAVVTYFMVLVQLKX-----------------
Mdom    -- VLGAVVTYFMVLVQLK------------------
Ccap    -- VLGAVVTYFMVLVQLK------------------
Csty    -- VLGAVVTYFMVLVQLK------------------
Agam    -- VLGAVVTYFMVLVQLK------------------
Cqui    -- VLGAVVTYFMVLVQLK------------------
Hobl    -- VLGATVTYFMVLVQLK------------------
Aqua    -- VLGAVVTYFMVLVQLK------------------
Dpon    -- VLGATVTYFMVLVQLK------------------
Tcas    -- VLGAVVTYFMVLVQLK------------------
Sinv    -- VLGAVVTYFMVLVQLK------------------
Amel    -- VLGAMVTYFMVLVQLK------------------
Mrot    -- VLGAMVTYFMVLVQLK------------------
Ccun    -- VLGAVVTYFMVLVQLK------------------
Csol    -- VLGAVVTYFMVLVQLK------------------
Nvit    -- VLGAVVTYFMVLVQLK------------------
Ccin    -- VLGAVVTYFMVLVQLK------------------
Mcin    -- VLGAVVTYFMVLEQL-------------------
Nlec    -- VVGAMVTYFMVLMQLG------------------
```

Altered functional properties of the Codling Moth Orco mutagenized in the intracellular loop-3

Yuriy V. Bobkov, William B. Walker III and Alberto Maria Cattaneo*

Supplementary Figure S1. Polypeptide sequence alignment of ICL-3 based on Topcons predictions among the insect representatives provided with a substitution of the ICL-3 histidine. Coloured lines and bars indicate motif A and critical residues based on models proposed by Miller and Tu[46]. Green line and bars: motif A and the most conserved residues according with the model of *An. gambiae* (G[T|N]ELTX[K|E][S|N]EX[V|I][A|S]DA[I|L|V]YSSPWY). Blue line and bars: motif A and the most conserved residues according with the model of *D. melanogaster* ([Q|E][L|T]F[P|L]YCYY[G|A][N|T]L[L|I|V]XXESEX[L|V]AXA[A|L][Y|F]SSNWY). Brown line and bars: motif A and the most conserved residues according with the model of *A. mellifera* (G[Q|E|D][I|L]LI[E|D][E|Q][S|C]X[N|E][I|V][G|A]NAVYMSNWY). Orange line: ICL-3 domain, note: BtabOrco, PsolOrco ICL-3 are predicted to start one residue upstream (Orange bar). Bold residues: amino acid substitutions at position of H417. Amino acids have been numbered starting from the first amino acid of the ICL-3 domain (G). Acronyms for insect names are based on Table 1.

```
        ..|....|.. ..|....|.. ..|....|.. ..|....|.. ..|....|.. ..|....|..
        -7    0       10        20        30        40        50
Cpom    QVFHFCIFGN RLIEESSSVM EAAYSCQWYD GSEEAKTFVQ IVCQQCQKAM SISGAKFF--
Phum    QVFVFCTYGN QLIEESSSVV EAAYSCQWYD GSEEAKTFVQ IVCQQCQKAL SISGAKFF--
Znev    QVFLFCFFGN RLIEESSSVM EAAYSCQWYD GSEEAKTFIQ IVCQQCQKAM SISGAKFFTV
Csec    QVFLFCFFGN RLIEESSSVM EAAYSCQWYD GSEEAKTFIQ IVCQQCQKAM SISGAKFFTV
Nlec    QVFLFCIFGN RLIEESSSVM DAAYSCQWYD GSEEAKTFVQ IVCQQCQKAM SVSGAK----
Tdom    QVFLFCIFGN DLIEESLSVM KAAYECPWYN GSEEAKTFIQ IVCQQCQRAL SISGAKFF--
Psic    QVFQFCYYGN QLIDESSSVL EAAYSCRWYD GTEEAKAFVQ IICQQCQKSM TISGAKFFTV
Btab    QVYAFCIYGN ELIEESSSVM EAAYDCRWYD GSEEAKTFVQ IVCQQCQKSL SISGAKFFTV
Dcit    QVFFFCFYGN ELIEQSSSVM EAAYSCSWYD GSEDAKTFVQ IVSQQCQKSL SITGSKF---
Psol    QIYTFCIYGN ELIDESSSVM EAAYSCSWYD GSEEAKTFVQ IVCQQCQKAL SVTGAKFF--

Dmel    QVFHFCIFGN RLIEESSSVM EAAYSCHWYD GSEEAKTFVQ IVCQQCQKAM SISGAKFF--

Agam    QVFLFCIFGN RLIEESSSVM EAAYSCHWYD GSEEAKTFVQ IVCQQCQKAM TISGAKFF--

Amel    QVFMLCIFGN RLIEESSSVM EAAYSCHWYD GSEEAKTFVQ IVCQQCQKAM SISGAKFF--
```

Altered functional properties of the Codling Moth Orco mutagenized in the intracellular loop-3

Yuriy V. Bobkov, William B. Walker III, Alberto Maria Cattaneo*

Supplementary Table S1. Percentages of fluorescent cells based on EBFP expression (EBFP +) and 800 µM VUAA1 application (Fluo4 +) from different HEK293A transfected with CpomOrco variants (Experiments 1, 2: CpomOrco, CpomOrco^{Q417H}) and co-transfecting CpomOrco variants with CpoOR6a subunit (Experiments 3, 4: CpomOrco+OR6a, CpomOrco^{Q417H}+OR6a).

Single subunit experiments

Cpom	Experiment 1				Experiment 2			
	Orco		Orco^{Q417H}		Orco		Orco^{Q417H}	
	Cells	%	Cells	%	Cells	%	Cells	%
Bright Field	342	-	537	-	348	-	546	-
EBFP +	140	**40,94**	207	**38,55**	115	**33,05**	222	**40,66**
Fluo4 +	106	**30,99**	99	**18,44**	122	**35,06**	93	**17,03**

Co-transfection experiments

Cpom	Experiment 3				Experiment 4			
	Orco+OR6a		Orco^{Q417H}+OR6a		Orco+OR6a		Orco^{Q417H}+OR6a	
	Cells	%	Cells	%	Cells	%	Cells	%
Bright Field	302	-	452	-	342	-	329	-
EBFP +	94	**31,13**	135	**29,87**	105	**30,70**	91	**27,66**
Fluo4 +	146	**48,34**	38	**8,41**	170	**49,71**	34	**10,33**
